# Effect of Cinnamon Oil on Quorum Sensing-Controlled Virulence Factors and Biofilm Formation in *Pseudomonas aeruginosa*

**DOI:** 10.1371/journal.pone.0135495

**Published:** 2015-08-11

**Authors:** Manmohit Kalia, Vivek Kumar Yadav, Pradeep Kumar Singh, Deepmala Sharma, Himanshu Pandey, Shahid Suhail Narvi, Vishnu Agarwal

**Affiliations:** 1 Department of Biotechnology, Motilal Nehru National Institute of Technology, Allahabad, India; 2 Department of Mathematics, National Institute of Technology, Raipur, India; 3 Faculty of Pharmaceutical Sciences, Sam Higginbottom Institute of Agriculture Technology & Sciences, Allahabad, India; 4 Department of Chemistry, Motilal Nehru National Institute of Technology, Allahabad, India; University Roma Tre, ITALY

## Abstract

Quorum sensing (QS) is a system of stimuli and responses in bacterial cells governed by their population density, through which they regulate genes that control virulence factors and biofilm formation. Despite considerable research on QS and the discovery of new antibiotics, QS-controlled biofilm formation by microorganisms in clinical settings has remained a problem because of nascent drug resistance, which requires screening of diverse compounds for anti-QS activities. Cinnamon is a dietary phytochemical that is traditionally used to remedy digestive problems and assorted contagions, which suggests that cinnamon might contain chemicals that can hinder the QS process. To test this hypothesis, the anti-QS activity of cinnamon oil against *P*. *aeruginosa* was tested, measured by the inhibition of biofilm formation and other QS-associated phenomena, including virulence factors such as pyocyanin, rhamnolipid, protease, alginate production, and swarming activity. To this end, multiple microscopy analyses, including light, scanning electron and confocal microscopy, revealed the ability of cinnamon oil to inhibit *P*. *aeruginosa* PAO1 biofilms and their accompanying extracellular polymeric substances. This work is the first to demonstrate that cinnamon oil can influence various QS-based phenomena in *P*. *aeruginosa* PAO1, including biofilm formation.

## Introduction

Quorum sensing (QS) is a communication system through which bacteria converse with one another and higher species [1]. QS is based on the synthesis and perception of specific chemical signals, often referred to as autoinducers, that accumulate in the growth medium during bacterial growth. When the concentration of autoinducers reaches a threshold value, corresponding to a certain population density, it alters the expression of genes. In many pathogenic bacteria, QS positively regulates genes responsible for virulence and biofilm formation [2]. Many gram-negative bacteria utilize acyl homoserine lactones (AHLs) as QS signaling molecules. These molecules may vary in the length and composition of the acyl side-chain, depending on the species. The genes involved in AHL-based QS systems are conserved among gram-negative bacteria and are referred to as *luxI*-like and *luxR*-like genes. The *luxI*-like gene encodes the enzyme required for the synthesis of an AHL, and the cognate *luxR*-like gene encodes an intracellular receptor of that specific AHL. Upon binding to the AHL signal, the receptor regulates the transcription of target genes [3]. *P*. *aeruginosa* is an opportunistic pathogen responsible for causing various infections, particularly in hosts with compromised immune systems. *P*. *aeruginosa* infections are frequently observed in patients suffering from cystic fibrosis [4]. QS is used by *P*. *aeruginosa* to produce virulence factors that assist in successfully establishing infections. *P*. *aeruginosa* has two AHL *lux*-like systems, *las*I/*las*R and *rhl*I/*rhl*R, which regulate the genes responsible for virulence factor production, and a third non-LuxI/LuxR QS system known as the *Pseudomonas* quinolone signal (PQS). In the *las*I/*las*R system, *las*I synthesizes 3-oxo-dodecanoylhomoserine lactone (3-oxo-C_12_HSL) [5]. When the concentration of 3-oxo-C_12_HSL passes a certain threshold, 3-oxo-C_12_HSL binds to the cytoplasmic receptor LasR and activates the expression of genes that produce virulence factors, such as proteases, elastases and exotoxin A [6]. In addition, the *las*I homolog *rhl*I is regulated by LasR-3-oxo-C_12_HSL. *rhl*I synthesizes butanoyl homoserine lactone (C_4_HSL), which, after passing a given threshold, binds to the receptor RhlR and activates certain target genes, including the genes responsible for the production of pyocyanin, elastases, siderophores, and rhamnolipids [7].

Biofilm formation is an additional QS-controlled activity in *P*. *aeruginosa*, even though biofilm formation in *P*. *aeruginosa* requires supplemental environmental signals along with QS-regulated factors, such as rhamnolipids and siderophores, and swarming motility. Bacteria growing in biofilm mode exhibit altered phenotypes that safeguard them from therapeutic treatment [8].

The rampant use of antibiotics to treat illnesses involving biofilms has resulted in the evolution of antibiotic-resistant microorganisms [9]. Because of this microbial antibiotic resistance, effective treatments are scarce, and there is a need to identify new treatment strategies. In this regard, QS is an exemplary target because it regulates assorted virulence factors [10].

Most QS inhibitors, both natural and synthetic, belong to a family of secondary metabolites known as furanones, which are structurally similar to AHLs and therefore bind to AHL receptors. However, QS inhibitors are incapable of activating AHL receptors. India has a wealth of ayurvedic medicines, and Indian people have long used ayurvedic herbs to treat contagions. Cinnamon is a dietary phytochemical that shows antimicrobial properties and is particularly significant given that dietary chemicals are deemed trustworthy and used habitually in daily life. Cinnamon is conventionally endorsed for treating digestive problems, including nausea, vomiting, and diarrhea [11]. Cinnamon oil is also used in several toothpastes as an antimicrobial substitute. The objective of this study was to investigate the ability of cinnamon oil to inhibit QS-mediated virulence factors and biofilm formation in *P*. *aeruginosa* PAO1.

## Materials and Methods

### Bacterial strains and culture conditions

This investigation used the bacterial strain *Chromobacterium violaceum* CV026, a double mini-Tn*5* mutant derived from ATCC31352 containing Kan^R^, which has been previously used to detect QS inhibitory activity. The strain was obtained from CECT, Spain. *P*. *aeruginosa* PAO1 was obtained from the MTCC (microbial type culture collection), IMTECH, Chandigarh. GFP-tagged *P*. *aeruginosa* PAO1 (GFP-PAO1) and *E*. *coli* pJN105L pSC11 were kindly provided by Dr. Peter Greenberg of the University of Washington. Cinnamon oil (Ceylon type), hexanoyl homoserine lactone (AHL), kanamycin, gentamycin, ampicillin, L-arabinose, and 3-oxo-C_12_HSL were purchased from Sigma Aldrich, India. AHL was dissolved in DMSO to obtain a stock solution of 0.5 mg/ml, and kanamycin was dissolved in MilliQ water to obtain a stock solution of 20 mg/ml. All strains were cultivated in Luria-Bertani (LB) medium (pH 7.0) and maintained at 37°C, except for *C*. *violaceum* CV026, which was grown at 30°C, and all strains were sub-cultured when the OD of the culture reached 1 at 600 nm. Tween-20 (SRL, India) was used as a surfactant for the uniform distribution of oil. PVC coupons were sterilized using the method described by Borucki *et al*. [12].

### Determination of the Minimum Inhibitory Concentration (MIC) of Cinnamon Oil

The MIC of cinnamon oil against *P*. *aeruginosa* PAO1 was determined following the guidelines of the NCCLS, USA (2006) [13, 14]. Briefly, an overnight culture of *P*. *aeruginosa* PAO1 (OD_600nm_ = 1) was grown for 24 h in LB medium mixed with different concentrations of cinnamon oil, ranging from 0.01 μl/ml to 1.5 μl/ml, in microcentrifuge tubes. The lowest concentration that completely inhibited growth was recorded as the MIC for cinnamon oil. Further experiments were conducted only at sub-MIC concentrations.

### Growth Curve Analysis

Growth curve analysis was performed to analyze the effect of sub-lethal concentrations of cinnamon oil on the different strains used in this study (i.e., *P*. *aeruginosa* PAO1, *C*. *violaceum* CV026, and *E coli* pJN105LpSC11). Overnight cultures of the tested strains were inoculated into 100 ml of LB broth supplemented with different concentrations of cinnamon oil (control, 0.1, 0.2, 0.4 and 0.6 μl/ml). The flasks were incubated at the optimal temperatures for the respective bacterial strains, and OD_600_ was monitored at 2 h intervals for up to 24 h.

### Violacein Inhibition Assay

Violacein was quantified using the method of Choo *et al*. [15]. Aliquots of 100 μl of an overnight culture of *C*. *violaceum* CV026 (adjusted to an OD of 1 at 600 nm) were added to the wells of a 96-well flat-bottom plate containing 100 μl of LB and incubated in the presence or absence of varying concentrations of cinnamon oil, ranging from 0.1 μl/ml to 0.6 μl/ml. The plate was incubated at 28°C for 16 h and then completely dried at 60°C. Next, DMSO (100 μl) was added to each well, and the 96-well plate was incubated at 30°C with shaking. DMSO alone was used as the negative control. The quantity of solubilized violacein was measured by determining the absorbance at 590 nm in an ELISA plate reader (Tecan, Sunrise).

### Antagonistic Activity of Cinnamon Oil Against *P*. *aeruginosa* QS

*E*. *coli* DH5α harboring pJN105L (containing the *lasR* gene) and pSC11 (P*lasI*::*lacZ*) was used as a bioreporter strain to determine the actual concentration of 3-oxo-C_12_HSL produced by *P*. *aeruginosa* PAO1 along the growth curve at every two hours in the culture supernatant by using the standard curve and to determine the antagonistic activities of different concentrations of cinnamon oil. Briefly crude bacterial supernatants were prepared by growing *P*. *aeruginosa* PAO1 in the presence of different concentrations of cinnamon oil and centrifuging 5 ml of the culture at 10,000g for 5 min. The supernatants were then filtered using a 0.2 μm filter into clean tubes. Overnight *E coli* pJN105LpSC11 culture were diluted with fresh LB medium to an OD_600_ of 1.0. In a sterilized 96-well plate, 100 μl of the sterile *P*. *aeruginosa* supernatant was mixed with 100 μl of 50- or 100-fold dilutions of *E coli* pJN105LpSC11. The *β*-galactosidase activity within the biosensor strain was measured using the Miller assay. [16, 17].

### Virulence Phenotype Assays

#### Pyocyanin Production

Supernatants from overnight cultures of *P*. *aeruginosa* PAO1 grown in the presence or absence of cinnamon oil were collected, and the pyocyanin pigment was extracted using chloroform, followed by 0.2 M HCl. This procedure yielded a pink-colored solution, and the absorbance was measured at 520 nm [18].

#### Swarming Assays

Swarming motility assays were performed based on a previously described method [19]. Briefly, overnight cultures of *P*. *aeruginosa* PAO1 were point inoculated onto swarm agar plates containing glucose (1%), bactoagar (0.5%), bactopeptone (0.6%), and yeast extract (0.2%) in the presence (0.2 μl/ml) or absence of cinnamon oil. The plates were incubated at 37°C in an upright position for 24 h.

#### Alginate Production

Alginate was extracted from both treated and untreated cultures of *P*. *aeruginosa* PAO1 using a method previously described by Knutson *et al*. [20]. Briefly, 500 μl of NaCl (1 M) was added to 500 μl of overnight culture and vortexed. The mixture was then centrifuged at 10,000 rpm for 20 min to remove residual alginate from the cell surface. Next, 500 μl of cetylpyridinium chloride was added to the supernatant, which was then inverted for mixing and centrifuged at 10,000 rpm for 10 min. The pellet was resuspended in 500 μl of chilled isopropanol for 10 min. This mixture was then re-centrifuged at 10,000 rpm for 10 min, and the pellet was resuspended overnight in 500 μl of 1 M NaCl. This suspension was used for the carbazole assay.

#### Azocasein Assay

Azocasein assays were performed to investigate the effect of cinnamon oil on protease production by *P*. *aeruginosa* PAO1. *P*. *aeruginosa* PAO1 was grown overnight at 37°C in the presence (0.1 μl/ml to 0.6 μl/ml) or absence of cinnamon oil. The cultures were centrifuged, and 1 ml of the supernatant was added to 0.5% azocasein in 0.1 M Tris buffer (pH 8); this mixture was incubated for 1 h at room temperature. The reaction was stopped by adding 10% trichloroacetic acid and incubating the reaction at 4°C for 20 min. The reaction mixture was subsequently centrifuged, and the supernatant was collected and mixed with an equal volume of 1 M NaOH. The absorbance was then measured at 420 nm [21].

#### Skim Milk Agar Assay

To determine the efficacy of cinnamon oil for inhibiting protease production, cultures were grown overnight in the presence or absence of cinnamon oil. These cultures were then centrifuged at 10,000 rpm for 15 min, and the supernatants were collected and loaded into the wells of skim milk agar plates. Protease activities were determined by measuring the diameter of the zone of clearance around each well after incubating the plates overnight at 37°C [22].

### Biofilm Inhibition Assay

Biofilms were grown under sublethal concentrations or in the absence of cinnamon oil in 96-well microtiter plates using a modified version of the Boddey *et al*. [23] method. Briefly, 100 μl of LB broth was added to each well of a 96-well microtiter plate, followed by the addition of oil to all except the control well. Then, 1 μl of bacterial culture was added to each well, followed by incubation at 37°C for 18 h. Thereafter, 1 μl of the growth culture from each well was transferred to a well in a new microtiter plate containing 100 μl of fresh LB, and this plate was incubated at 37°C for 24 h. After incubation, the supernatant was discarded, and the wells were washed with phosphate-buffered saline and stained with 200 μl of 0.4% crystal violet. The stain was discarded, and the wells were washed with distilled water before adding DMSO to solubilize the crystal violet. The plate was then read spectrophotometrically at 590 nm in an ELISA reader.

### Microscopy Analysis

The effect of cinnamon oil on biofilm formation was analyzed as described previously by Musthafa *et*. *al*. [24]. Overnight cultures of *P*. *aeruginosa* PAO1 (adjusted to an OD of 1 at 600 nm) were added to fresh media containing sterilized cover slips and cinnamon oil in 50 ml centrifuge tubes, followed by incubation for 16 h. After incubation, the cover slips were rinsed with phosphate-buffered saline to remove loosely adhered cells. The biofilm that adhered to the coverslip was then stained with a 0.4% crystal violet solution and visualized under a light microscope (Olympus) at 100X magnification.

#### Scanning Electron Microscopy

SEM was performed following the method of Hawser *et al*. [25]. Briefly, *P*. *aeruginosa* PAO1 biofilms that formed on pieces of PVC (1.0 cm^2^) in the presence or absence of cinnamon oil were fixed with 2.5% (v/v) glutaraldehyde in PBS for 5 h at room temperature. The samples were then washed with PBS and dehydrated in an ethanol series (30, 50, 70, 90, and 100%). All of the samples were subsequently dried overnight, gold coated and viewed under SEM (Zeiss EVO MA 5).

#### Confocal Laser Scanning Microscopy

Confocal laser scanning microscopy (CLSM) analysis of the *P*. *aeruginosa* PAO1 and GFP-PAO1 biofilms was performed as described by Zhao *et al*. [26]. For the CLSM analysis, GFP-PAO1 and *P*. *aeruginosa* PAO1 were allowed to grow on a PVC surface in the presence or absence of cinnamon oil. After 16 h, the resulting biofilms were fixed with 5% glutaraldehyde in PBS for 30 min and then stained with 1 μg/ml DAPI (GFP-PAO1) or 0.5 μg/ml FITC-conA (*P*. *aeruginosa* PAO1). CLSM was performed using a Leica TCS SP5 confocal microscope, and the images were captured at 10X magnification.

### Statistical Analysis

All experiments were performed in triplicate, and the obtained results are expressed as the mean values and standard deviations. Differences between the controls and tests were analyzed using Student’s t-test (two-tailed) for unpaired samples, and only results at p<0.05 were considered significant.

## Results

### Effect of Cinnamon Oil on *P*. *aeruginosa* Growth

The MICs were calculated using the micro-broth dilution method with the concentration of cinnamon oil varying from 0.01 μl/ml to 1.5 μl/ml. The MIC of cinnamon oil for *P*. *aeruginosa* PAO1 was 1 μl/ml, and further experiments were conducted at a sub-lethal concentration.

**Fig 1:**
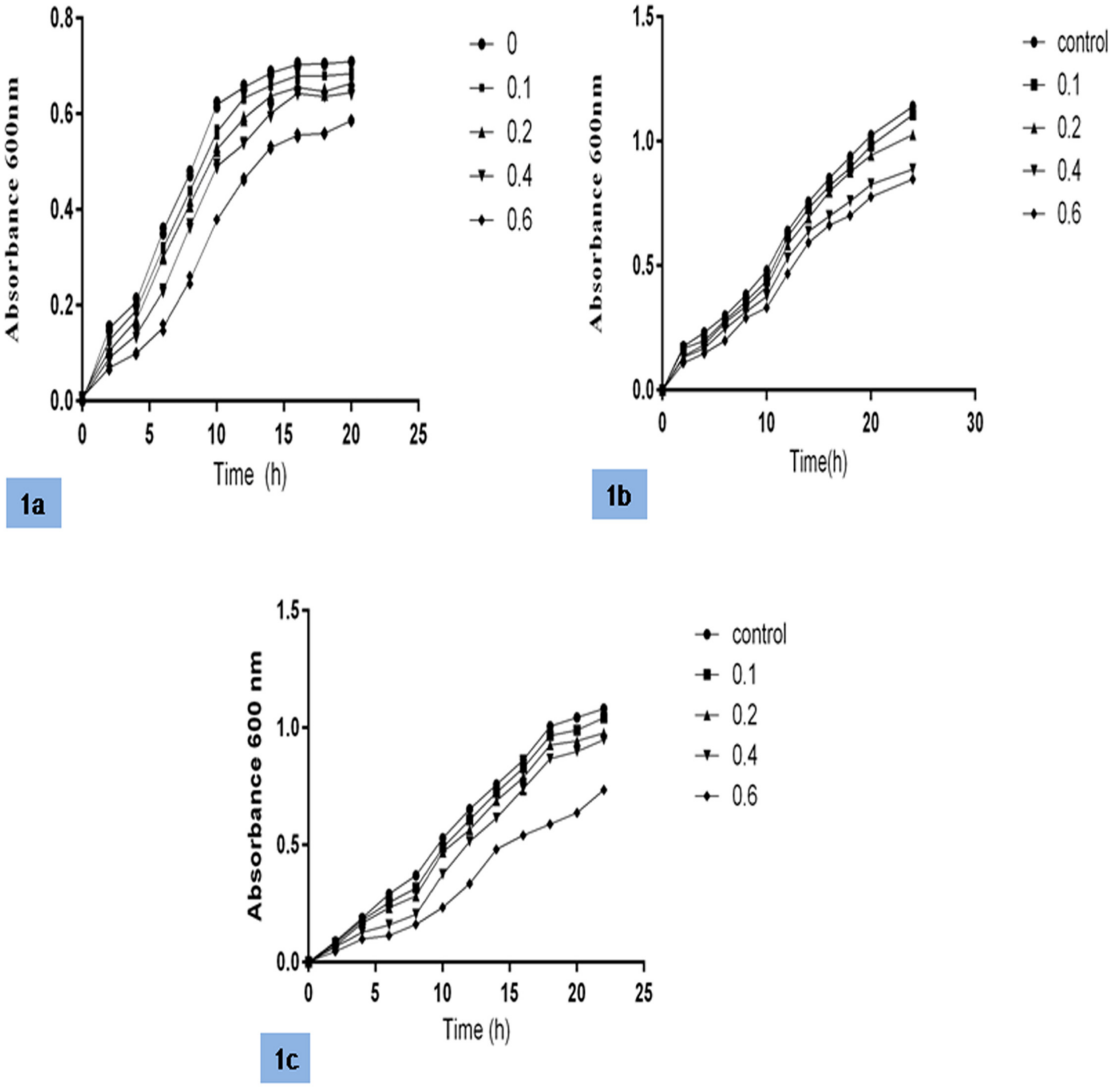
Growth curves of *P*. *aeruginosa* PAO1 (1a), *C*. *violaceum* CV026 (1b) and *E coli* pJN105LpSC11 (1c) at a sub-lethal concentration of cinnamon oil, plotted with respect to the controls (no cinnamon oil).

The effects of a sub-lethal concentration of cinnamon oil on the growth curves of *P*. *aeruginosa* PAO1, *C*. *violaceum* CV026 and *E coli* pJN105LpSC11 were analyzed. The results revealed that although lower concentrations of oil (0.1 and 0.2 μl/ml) did not significantly affect the growth curves of the different strains, concentrations higher than 0.2 μl/ml resulted in a reduced growth rate and may have affected other bacterial processes in addition to QS (Fig 1A–1C).

### Effect of Cinnamon Oil on AHL Production

**Fig 2:**
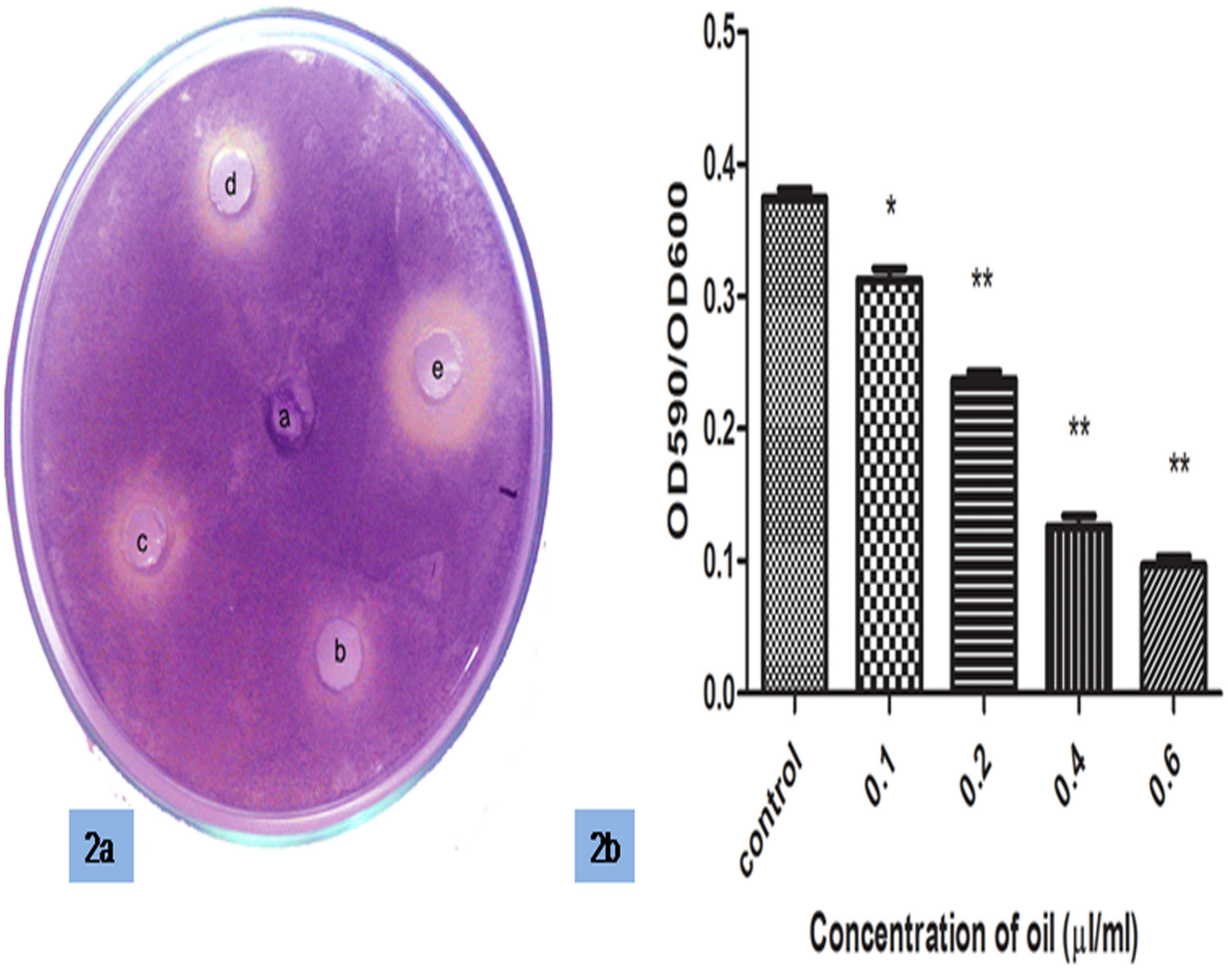
QS inhibition of *C*. *violaceum* CV026 by cinnamon oil. (a) The zone of inhibition with increasing concentrations of oil (a-control, b-0.1, c-0.2, d-0.4, e-0.6 μl/ml). (b) A decrease in violacein production was observed. The color change was measured at 590 nm. The error bars indicate the standard deviations of three measurements, and the data were normalized according to the OD_600_ of CV026.*, P<0.05 compared with the control. **, P<0.001 compared with the control.

Violacein production was qualitatively estimated in the mutant strain *C*. *violaceum* CV026 (which produces violacein when AHL is supplied exogenously) in the presence of increasing concentrations of cinnamon oil. Violacein production decreased with increasing concentrations of oil (Fig 2a). In the quantitative inhibition assay, violacein pigment production was reduced by approximately 36% at a concentration 0.2 μl/ml cinnamon oil. Violacein pigment production was decreased with an increasing concentration of oil, with approximately 78% inhibition of violacein production observed at a concentration of 0.6 μl/ml. This effect possibly occurred due to a combination of QS inhibition and a decreased growth rate, as shown in Fig 2b.

**Fig 3:**
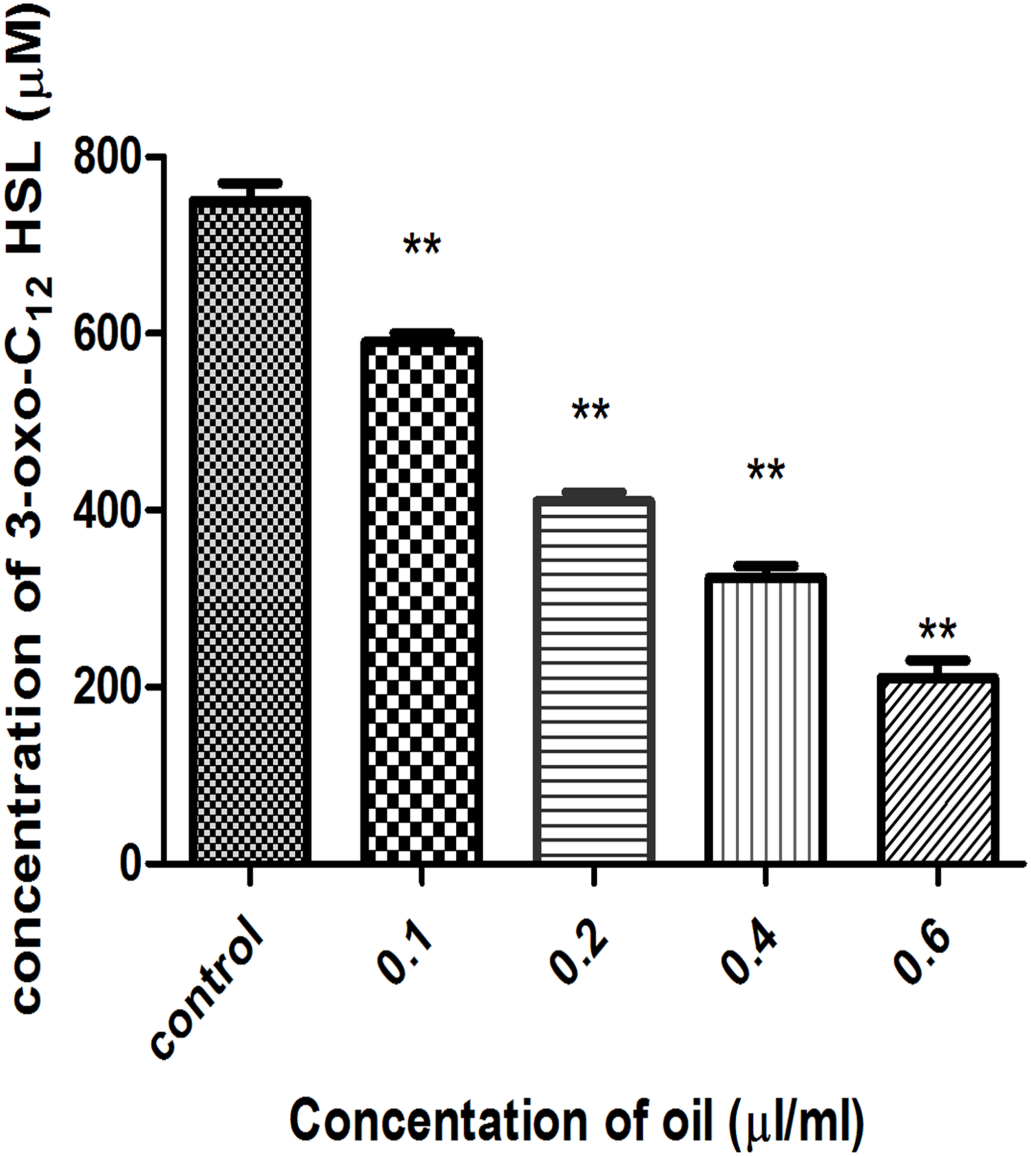
Effects of different concentrations of cinnamon oil (control, 0.1, 0.2, 0.4, 0.6 μl/ml) on the concentration of 3-oxo-C_12_HSL produced by *P*. *aeruginosa* PAO1grown up to 24h.

Sub-lethal concentrations of cinnamon oil were assayed for their QSI activity against *Pseudomonas aeruginosa* using the *E*. *coli* pJN105LpSC11 reporter strain. 3–Oxo-C_12_ HSL levels measured in the PAO1 culture supernatant decreased in the presence of increasing concentrations of cinnamon oil (Fig 3). The data showed a maximum reduction of the production of 3–oxo-C_12_ HSL of approximately 38% in response to a 0.2 μl/ml concentration of the oil. A further reduction was observed at higher concentrations, possibly due to the combined effect of a reduced growth rate and QS inhibition (S1 and S2 Figs).

### Effect of Cinnamon Oil on *P*. *aeruginosa* Virulence Factors

**Fig 4:**
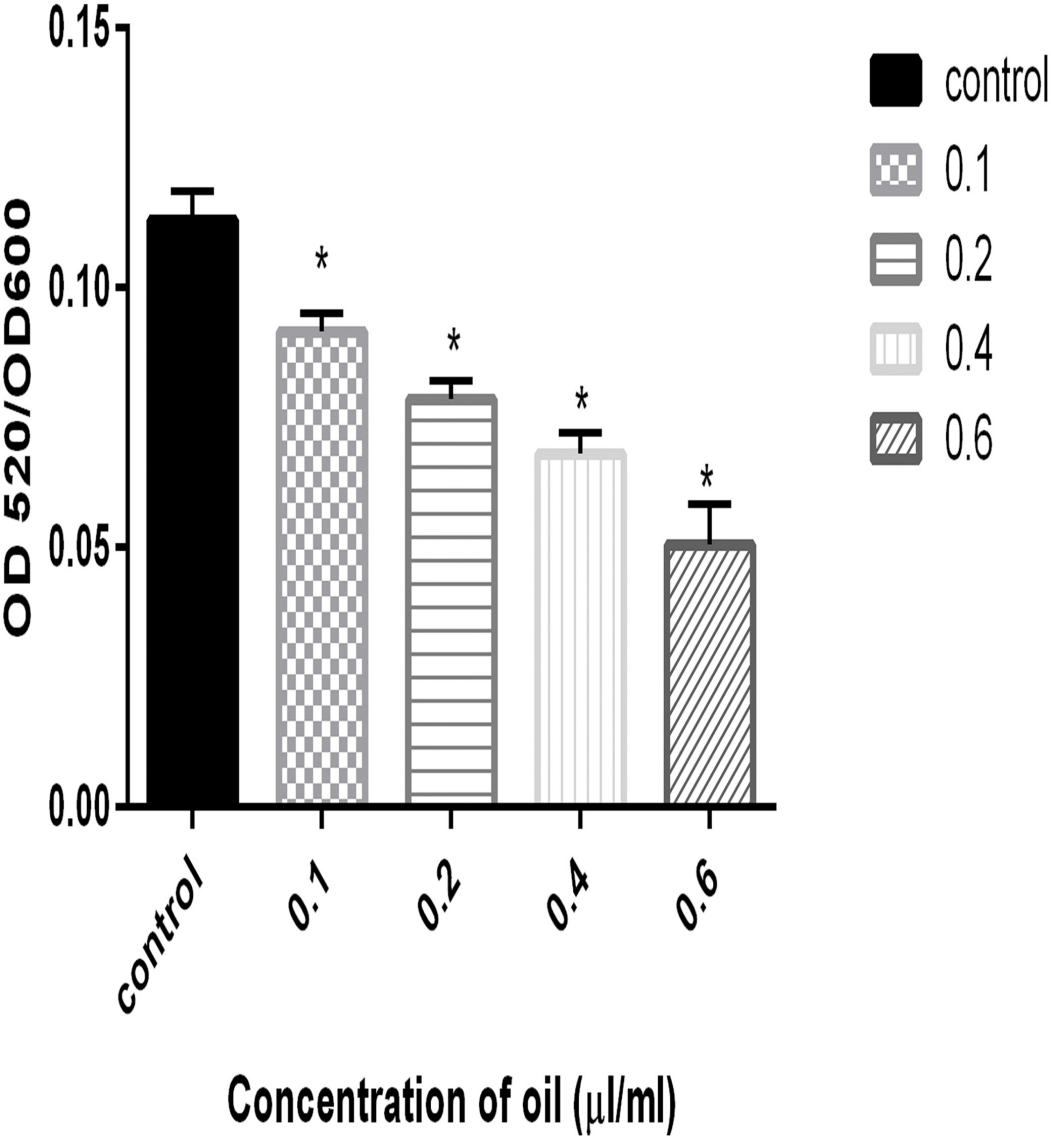
Effects of different concentration (0.1–0.6 μl/ml) of cinnamon oil on the production of pyocyanin. Error bars indicate the standard deviations for three measurements, and the data were normalized according to the OD_600_.*, P<0.05 compared with the control.

*P*. *aeruginosa* PAO1 produces a green-colored cytotoxic factor that plays an important role during *P*. *aeruginosa* host infections. Genes responsible for the synthesis and secretion of the pyocyanin pigment are regulated by QS. The effects of different concentrations of cinnamon oil on the ability of the bacteria to synthesize and secrete pyocyanin were investigated. The results indicated that the extent of pyocyanin formation decreased with increasing concentrations of cinnamon oil, showing a reduction to approximately 22% at a concentration of 0.2 μl/ml compared with the control, as shown in Fig 4.

**Fig 5:**
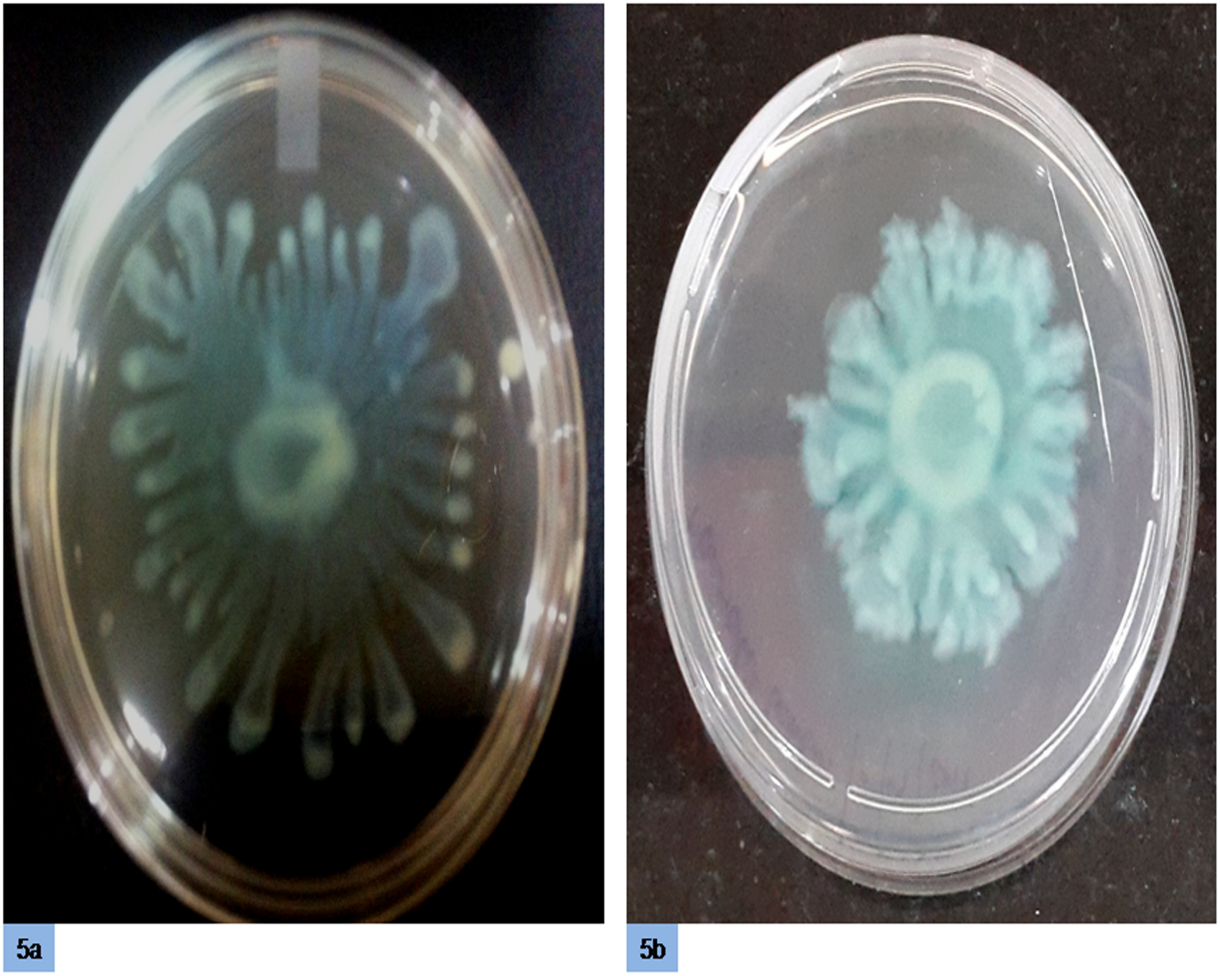
Swarming motility of *P*. *aeruginosa* PAO1. (a) Untreated (b) treated with 0.2 μl/ml cinnamon oil.

Swarming usually occurs on semi-solid agar. In this study, 0.5% swarming agar was optimized. The swarming motility of *P*. *aeruginosa* PAO1 was evaluated by spotting a culture of *P*. *aeruginosa* PAO1 on swarming agar plates with or without cinnamon oil and measuring the lengths of dendrites on these plates. Swarming inhibition against *P*. *aeruginosa* PAO1 could be observed on swarming agar containing 0.2 μl/ml cinnamon oil (Fig 5).

**Fig 6:**
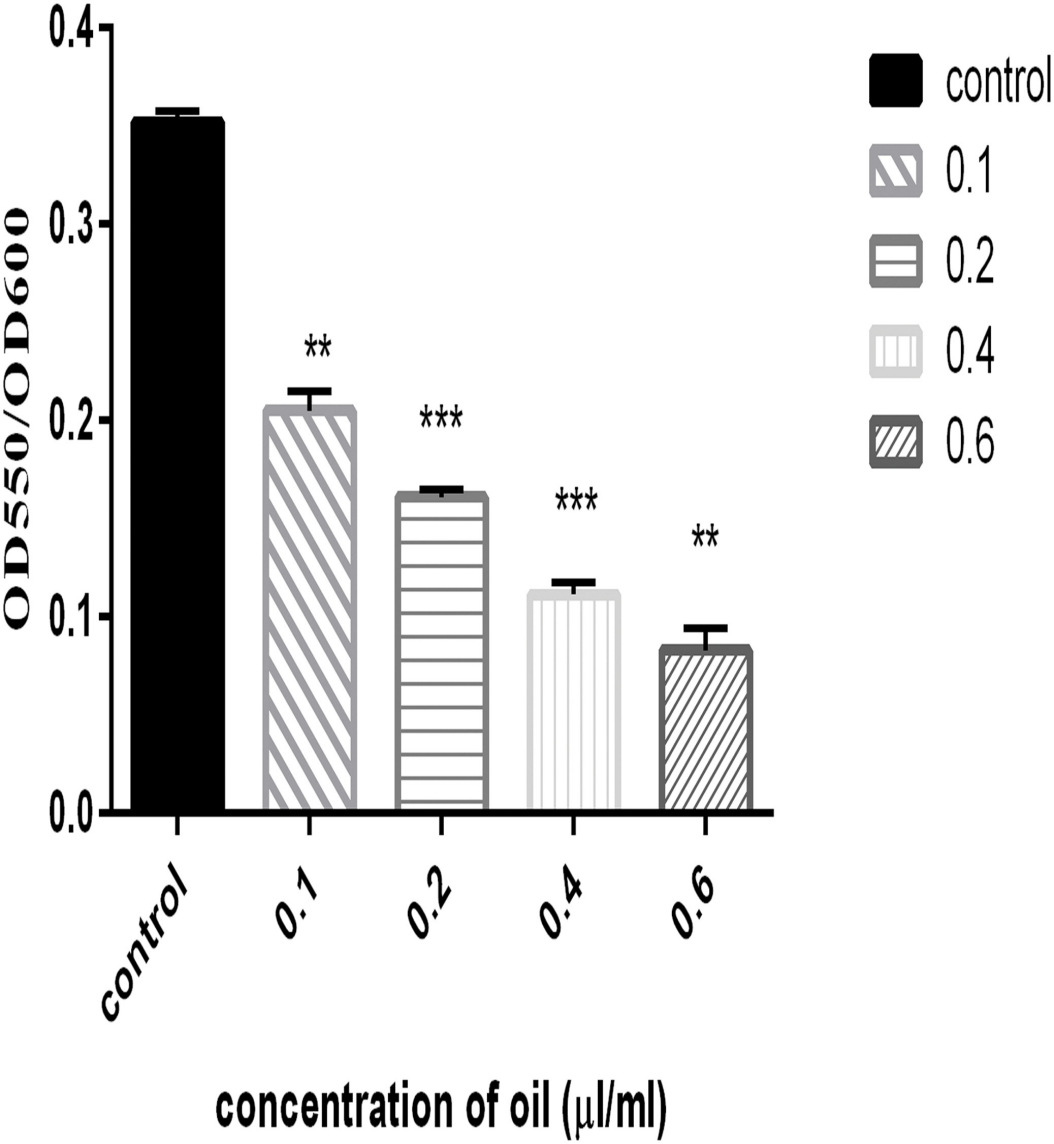
Effect of cinnamon oil on the production of alginate by *P*. *aeruginosa* PAO1. Error bars indicate the standard deviation of three measurements, and the data were normalized according to the OD_600_.**, P<0.001 compared with the control.***,P<0.0001 compared with the control.

Alginate is one of the major constituents of the EPS secreted by *P*. *aeruginosa* PAO1 during biofilm formation. The efficacy of cinnamon oil in reducing alginate production was examined, and alginate production was found to be reduced significantly with increasing concentrations of cinnamon oil. There was a 54% reduction in alginate production at a concentration of 0.2 μl/ml in comparison with the control (Fig 6).

**Fig 7:**
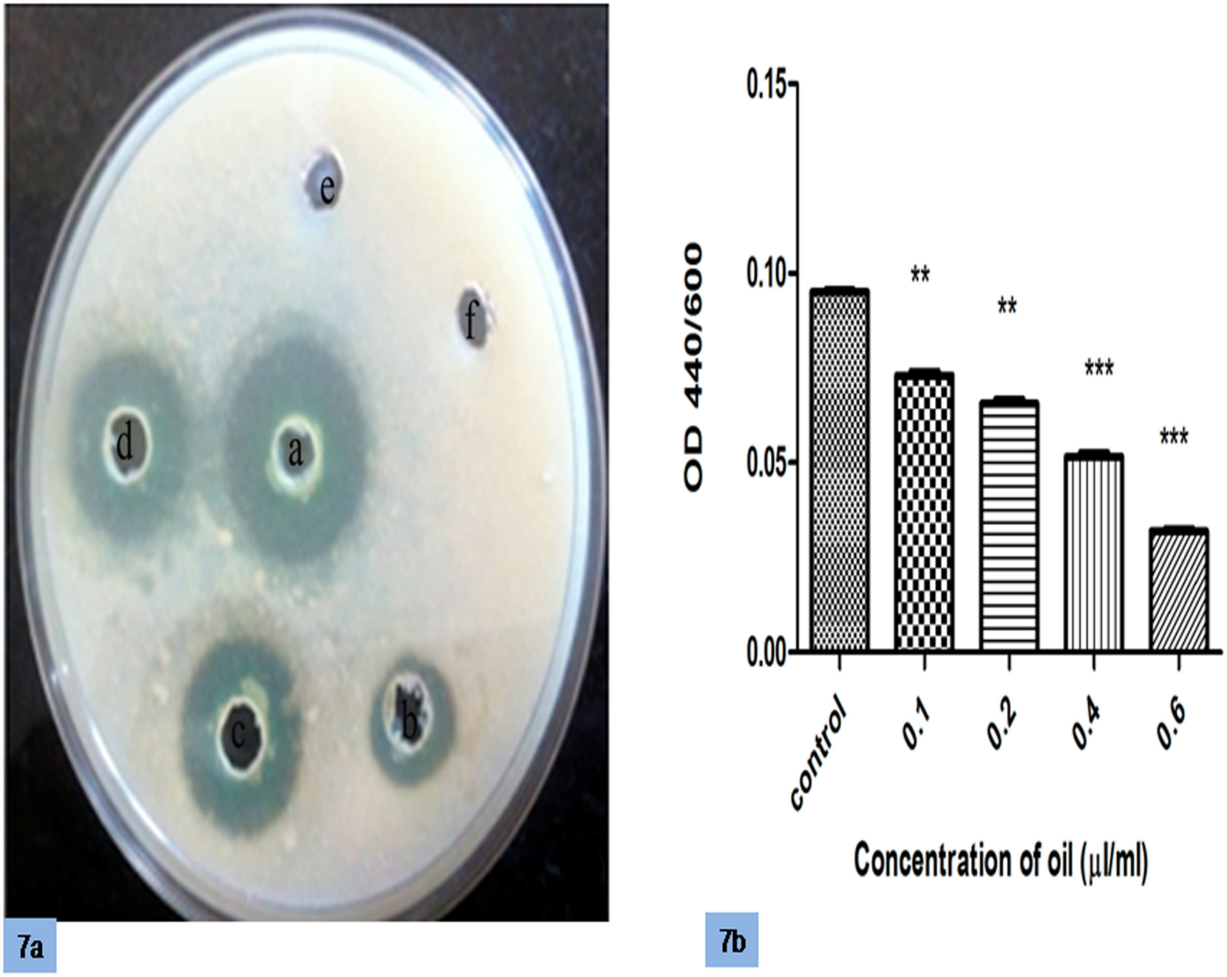
(a) Detection of the inhibition of protease production by cinnamon oil on skimmed milk agar plates (a-control, b-0.1, c-0.2, d-0.4, e-0.6 μl/ml). (b) Effects of different concentrations of cinnamon oil on the production of protease. Error bars indicate the standard deviations of three measurements, and the data were normalized according to the OD_600_. **, P<0.001 compared with the control. *** P<0.0001 compared with the control.

Total protease activity was measured using two assays: the azocasein assay and the skimmed milk agar plate assay. In the azocasein assay, the protease activities of untreated cultures and cultures grown in the presence of increasing concentrations of oil were estimated. A decrease in protease activity of approximately 22% was observed after treatment with 0.2 μl/ml cinnamon oil compared with the control, and there was a further decrease in protease production with an increase in the concentration of oil, possibly due to the combined effect of a reduced growth rate and QS inhibition. In the skim milk agar assay, the supernatants from treated and untreated cultures were added to the wells of a skim milk agar plate, and the clearance zones were shown to decrease with higher concentrations of oil (Fig 7a and 7b).

### Effect of Cinnamon oil on Biofilm Formation

**Fig 8:**
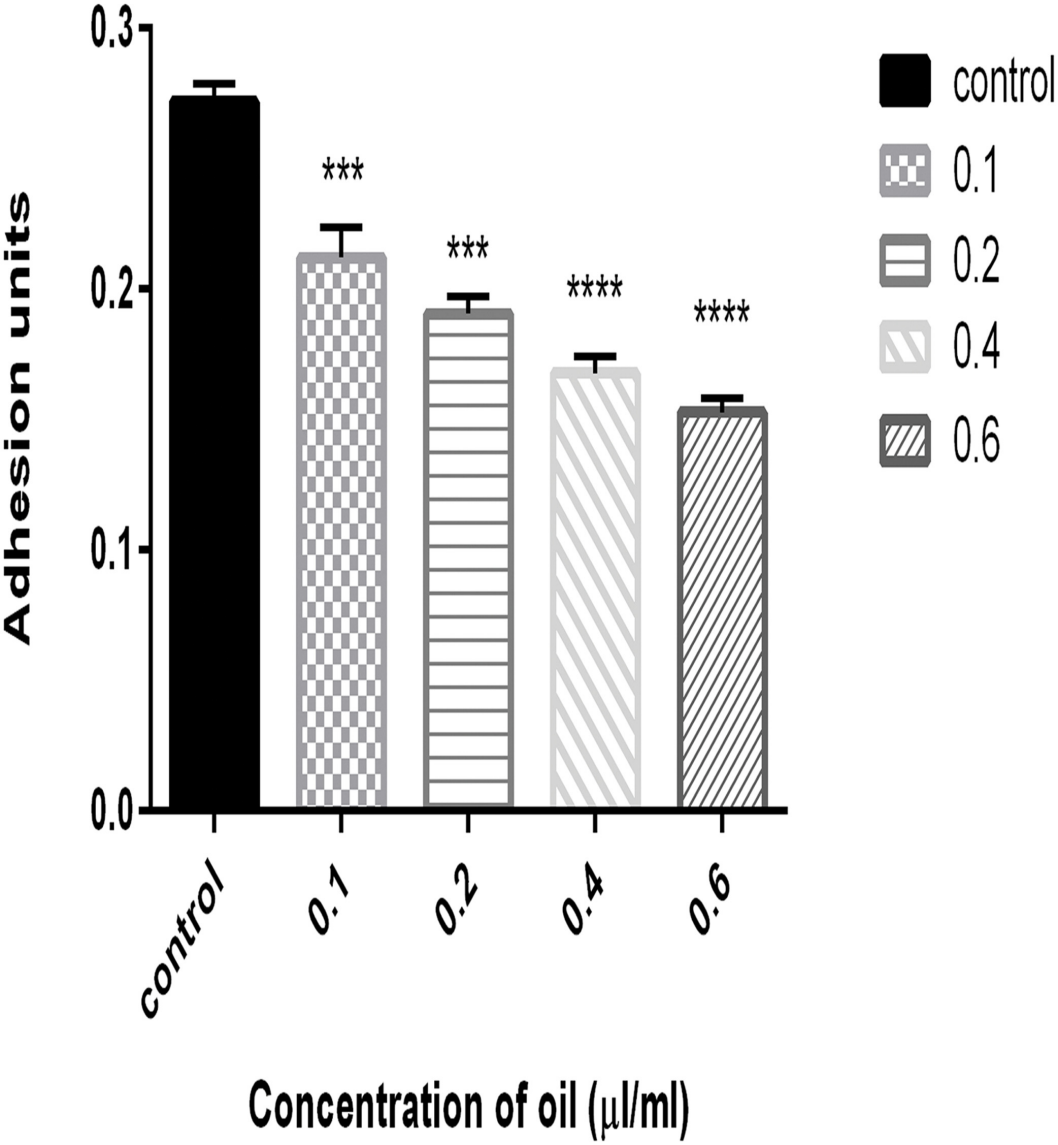
Quantification of PAO1 biofilms in the presence of various concentrations of cinnamon oil. The biofilms were quantified after 24 h of incubation and are represented as adhesion units (OD590/OD600). The error bars indicate the standard deviations of three measurements, and the data were normalized according to the OD_600_. ***, P<0.0001 compared with the control. ****, P<0.00001 compared with the control.

Biofilm quantification was performed to investigate the effect of cinnamon oil on biofilm formation. In a conventional crystal violet binding assay, biofilm formation was observed to be reduced by approximately 31% in the presence of 0.2 μl/ml cinnamon oil compared with the control (Fig 8).

### Microscopy Analysis of Biofilms

To clearly demonstrate the effect of cinnamon oil on biofilm, microscopic examinations were performed in the presence of high sub-lethal concentrations of cinnamon oil, which strongly inhibits the growth rate of planktonic cells. The microscopy analysis revealed changes in various parameters, including the biofilm-associated surface topology, structure, integrity, extra polymeric substrate (EPS) production, cell viability and DNA content.

**Fig 9:**
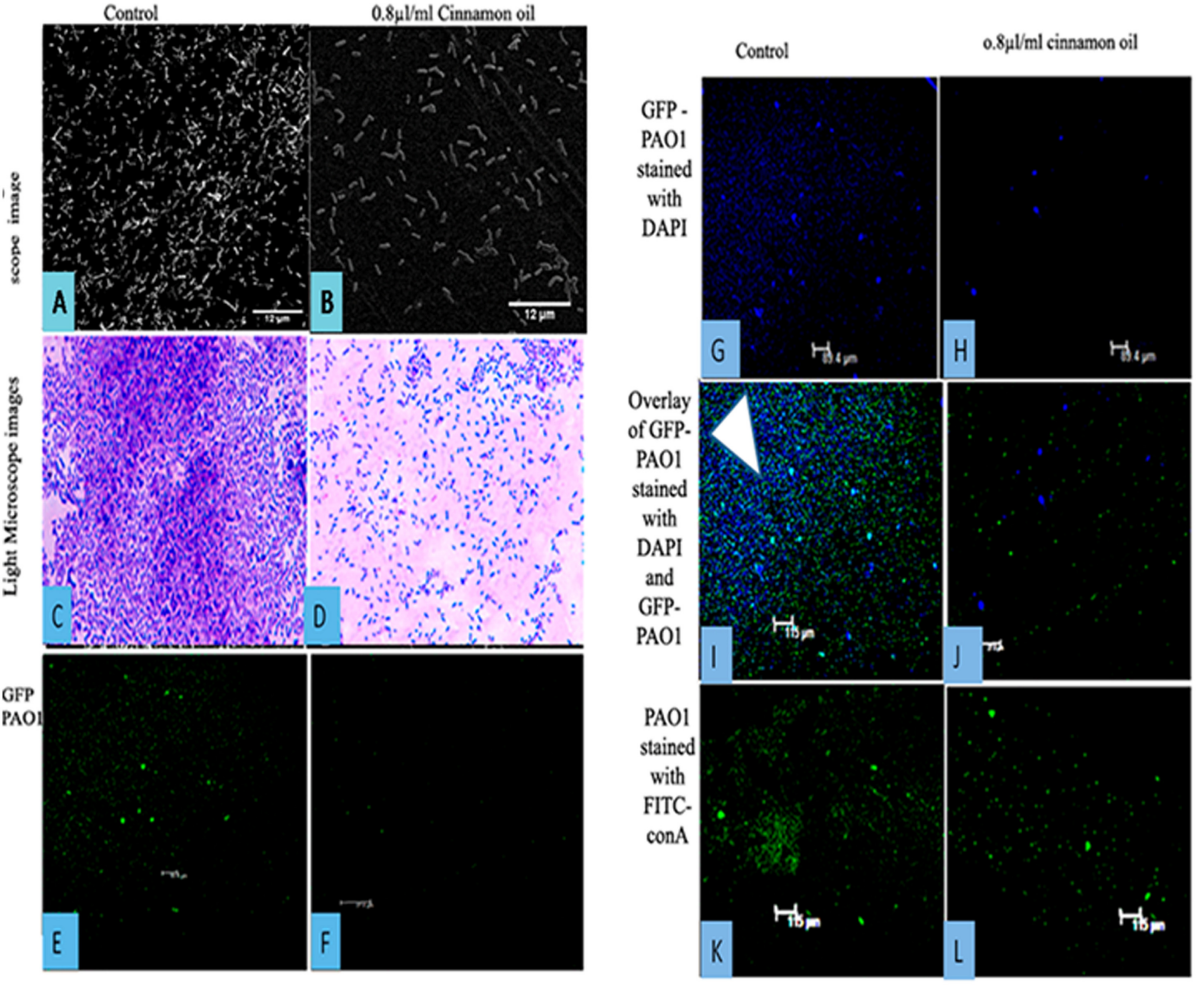
Microscopy images of *P*. *aeruginosa* PAO1 biofilms that formed with (right) 0.8 μl/ml and without (left) cinnamon oil. Scanning electron microscopy (a-b) and light microscopy images (c-d). Inhibition of GFP-PAO1 biofilm formation (e-f). Significant reduction in the DNA content of the GFP-PAO1 biofilm (g-h). Overlay of (e-f, g-h) images displaying the reduction in extracellular DNA associated with the GFP-PAO1 biofilm (i-j). In the image (i), extracellular DNA is indicated with an arrowhead. A reduction in EPS production was observed after staining *P*. *aeruginosa* PAO1 with FITC-conA. This effect is evident from the scattered appearance of the cells in the treated samples (k-l).

Visualization of the biofilms through SEM revealed the efficacy of cinnamon oil as a potent biofilm inhibitor, and the images of treated samples displayed a scattered appearance compared with the 24 h *P*. *aeruginosa* PAO1 control colonization. The sessile cells associated with the surface were scattered, and cell clusters were rarely visible because of poor cohesiveness and subsequent adherence. The integrity of the biofilms in terms of EPS production was also limited in the treated samples (Fig 9a and 9b). SEM is a destructive technique that relies on rigorous sample preparation and dehydration, which may result in unexpected or spurious results. To circumvent this problem, samples were examined using a light microscope at 100X after staining with crystal violet, and the results were similar to those obtained through SEM analysis (Fig 9c and 9d). To visualize EPS and extracellular DNA contents, which are integral components of biofilm structure and integrity, the samples were visualized using the fluorescent dyes FITC-ConA and DAPI. *P*. *aeruginosa* PAO1 stained with FITC-conA and PAO1-GFP stained with DAPI were used to avoid overlapping fluorescent spectra to visualize the GFP-expressing PAO1 cells to determine viability and EPS production using a mannose-binding dye (FITC-ConA). The confocal microscopy data revealed scattered cells and reduced EPS production. A decrease in the extracellular DNA content was also observed compared with the control (Fig 9e–9l).

## Discussion

QS is a system that is widely used by pathogenic bacterial species to regulate the expression of virulence factors associated with different pathogenic phenotypes through adherence and biofilm formation [26]. QS inhibition has emerged as a promising target in a wide variety of bacterial infections following the emergence of antibiotic-resistant phenotypes, which has resulted in a search for new therapeutic alternatives. These therapeutic alternatives must be active against a broad spectrum of bacteria to avoid provoking undesirable or immunological responses and should have the ability to treat a wide variety of targeted diseases. For decades, plant products and their derivatives have predominated infection therapy. Furanone derivatives from plants in South Florida [27] and garlic [28] as well as other dietary phytochemicals, such as *Curcuma longa* [29], caffeine [30] and vanilla extract [14], are considered to be potent inhibitors of QS and its regulatory factors. This work aimed to investigate the potential of cinnamon oil, a dietary supplement used worldwide, to inhibit QS and the associated virulence factors of pathogenic *P*. *aeruginosa* PAO1.

Cinnamon oil is a plant-derived oil, and its composition may vary depending on the bulk, the species of plant used, and the method of preparation. Cinnamon oil has been reported to exhibit potential antimicrobial activities against wide range of microorganisms [31, 32]. We used sub-lethal concentrations of cinnamon oil that are insufficient to neutralize microbes. No significant differences in growth patterns were observed between control and oil-treated *P*. *aeruginosa* PAO1, *C*. *violaceum* CV026 or *E coli* pJN105LpSC11 at the lower tested concentrations, 0.1 and 0.2 μl/ml, in the growth curve analysis. The growth curve data showed that cinnamon oil at low concentrations (0.1 and 0.2 μl/ml) inhibits QS, while the remaining concentrations tested in the study affect both QS and the growth rate. Our results are consistent with the findings of Kim *et al*., who reported decreases in biofilm formation and toxin production in the presence of different sub-lethal concentrations of cinnamaldehyde. Kim *et al*. also demonstrated a delay in reaching log phase in all treated samples [33].

However, cinnamaldehyde, which has been reported to be the major component of cinnamon oil, has been cited for its anti-QS activity in the *Vibrio harveyi* system by Brackman *et al*. [34]. These investigators reported that cinnamaldehyde and its derivatives are potentially useful anti-pathogenic compounds for the treatment of vibriosis and interfere with AI-2-based QS by decreasing the ability of LuxR to bind to its target promoter sequence [35]. Similarly, Yap PS *et al*., 2014 reported the membrane-permeabilizing effects of *Cinnamon verum* oil on treated cultures of multi-drug-resistant *Escherichia coli* J53 R1, further demonstrating the anti-QS activity of this oil [36]. We showed that complete cinnamon oil is active against QS-based virulence factors in *P*. *aeruginosa* PAO1, in which the QS molecules have been reported to be 3-oxo-C_12_HSL and C_4_HSL, rather than 3-hydroxy-butanoyl homoserine lactone, as in *V*. *harveyi*. In addition, we used Ceylon-type whole cinnamon oil, which has been reported to contain eugenol as its major constituent rather than cinnamaldehyde [37]. The presence of many major and minor components in cinnamon oil, including cinnamaldehyde and eugenol, suggests that the QSI activity of cinnamon oil may lie in its constituents either individually or synergistically. However, further experiments are required to address this question.

QS was measured both qualitatively and quantitatively using the mini-Tn*5* transposon mutant *C*. *violaceum* CV026, which produces a purple-colored pigment, violacein, that is regulated by the Cvi/R QS system and responds to AHL [38]. Our data showed decreased violacein production when AHL was added exogenously, as observed through halo zone formation. This finding supports the results of Khan *et al*. [39], who examined the anti-QS activities of 21 essential oils and observed QS reduction by clove oil, followed by *Cinnamon verum* oil, using the biosensor strain *C*. *violaceum* CV026. To clearly demonstrate the QSI activity of cinnamon oil against *P*. *aeruginosa* PAO1, an *E coli* DH5α strain harboring pJN105L (containing *lasR*) and pSC11 (P*lasI*::*lacZ*) was used as a bioreporter strain, and it was observed that the 3–oxo-C_12_ HSL levels measured in the PAO1 culture supernatants decreased in the presence of increasing concentrations of cinnamon oil, possibly due to the combined effect of QS inhibition and growth rate inhibition.

The effect of cinnamon oil on *P*. *aeruginosa* PAO1 was further evaluated by performing assays for QS-controlled virulence factors. Swarming is a major virulence factor in *P*. *aeruginosa* PAO1 that helps it colonize surfaces. Swarming movements are characterized by the movement of bacteria in a semisolid medium as they form tendrils to search for nutrients in nutrient-deficient medium; swarming helps *P*. *aeruginosa* PAO1 infection to spread. The correlation between biofilm formation and swarming motility remains unknown because of conflicting results in the literature. Rampioni *et al*., 2009 reported that the *rsaL* mutant of *P*. *aeruginosa* PAO1 overproduced virulence factors, including exhibiting increased swarming motility with reduced adhesion [40] whereas Kim *et al*., 2015 showed that cinnamon bark oil and cinnamaldehyde significantly reduced both *P*. *aeruginosa* PAO1 biofilms and swarming motility [41].

In this study, the swarming motility of *P*. *aeruginosa* PAO1 was effectively reduced in the presence of cinnamon oil, as indicated by the decreased length of tendrils compared with the control, which displayed closely spaced tendrils. These results are consistent with Packiavathy *et al*. [29], who reported inhibition of swarming motility by *Curcuma longa*; however, an enhancement of swarming motility by ginger has also been reported [42].

Pyocyanin is another major virulence factor produced by *P*. *aeruginosa* PAO1. Pyocyanin is a phenazine derivative that generates reactive oxygen species by oxidizing reduced glutathione in cells and simultaneously reducing oxygen [26]. In this study, cinnamon oil was shown to reduce pyocyanin production at increasing concentrations. Alginate is an important component of extracellular polysaccharide that helps to maintain biofilm structure and integrity and confers resistance to antimicrobials by preventing their entry. In this investigation, increased concentrations of cinnamon oil resulted in decreased alginate production, which is consistent with a previous report by Owlia *et al*. [43] showing that the oil of *Matricaria chamonilla* inhibits alginate production. Cinnamon oil decreased biofilm formation at sublethal concentrations, which was further confirmed by light microscopy, SEM and CLSM. Due to the resistant nature of biofilms, the microscopy analysis of *P*. *aeruginosa* PAO1 biofilm was performed using a high sublethal concentration (0.8 μl/ml) of cinnamon oil which has inhibitory effect on *P*. *aeruginosa* PAO1 growth rate. The biofilm microscopy data supported the quantitative findings. In this study, CLSM was performed to quantify EPS and extracellular DNA contents by staining *P*. *aeruginosa* PAO1 and GFP-PAO1 with FITC-ConA and DAPI, respectively. DAPI is a fluorescent dye that binds to AT-rich regions of DNA, whereas FITC-ConA binds to the mannose and glucose moieties of biofilm-associated EPS. These dyes were used to quantify DNA and EPS, respectively, to avoid obtaining overlapping fluorescent spectra. Many researchers have reported antibiofilm activities of cinnamon essential oil. Kerekes *et al*., 2013 used cinnamon oil against biofilms arising from mixed cultures of *E*. *coli* and *P*. *putida* and claimed to achieve complete inhibition [44]. Kim *et al*., 2015 showed that a 0.05% (v/v) concentration of cinnamon bark oil and cinnamaldehyde significantly reduced *P*. *aeruginosa* PAO1 biofilms [33]. Bouhdid *et al*., 2010 also demonstrated the effect of *Cinnamon verum* essential oil against *P*. *aeruginosa* ATCC 27853 planktonic cultures and claimed that it damaged the bacterial cellular membrane and induced cell death through collapse of the membrane potential [45]. These findings demonstrate that cinnamon oils of different origin may vary in their compositions and modes of action against a planktonic or biofilm mode of growth.

In this study, we have, for the first time, obtained preliminary microscopy data showing a reduction in biofilm-associated extracellular DNA contents and EPS in response to cinnamon oil, suggesting that whole cinnamon oil not only inhibits QS at a concentrationof approximately 0.2 μl/ml but also inhibits biofilm formation by reducing the associated extracellular DNA content and EPS, which are well-demonstrated components of biofilm functionality [46]. These results support the findings of a study by Hentzer and Givskov [47], in which furanones from *D*. *pulchra* [27] and garlic [28] caused a remarkable reduction of the EPS production and thickness of biofilms.

This study clearly demonstrates the ability of cinnamon oil to inhibit QS at lower concentrations (0.1–0.2 μl/ml), while both QS and the growth rate are affected at higher concentrations, possibly due partly to QS inhibition and partly to other unknown effects that together cause inhibition of the growth rate. Our findings provide a comprehensive overview of the inhibition of *P*. *aeruginosa* PAO1-associated virulence factors by oil from the popular dietary phytochemical cinnamon.

## Supporting Information

S1 Fig Production of 3-oxo-C_12_HSLduring growth curve in response to different concentrations of cinnamon oil. (TIF)

S2 Fig Standard curve of 3-oxo-C_12_HSL. (TIF)
