# Supplementary figures and images for: Effect of Cinnamon Oil on Quorum Sensing-Controlled Virulence Factors and Biofilm Formation in Pseudomonas aeruginosa
Source: PLoS One. 2015 Aug 11;10(8):e0135495. doi: 10.1371/journal.pone.0135495 (PMC4532483; doi:10.1371/journal.pone.0135495)

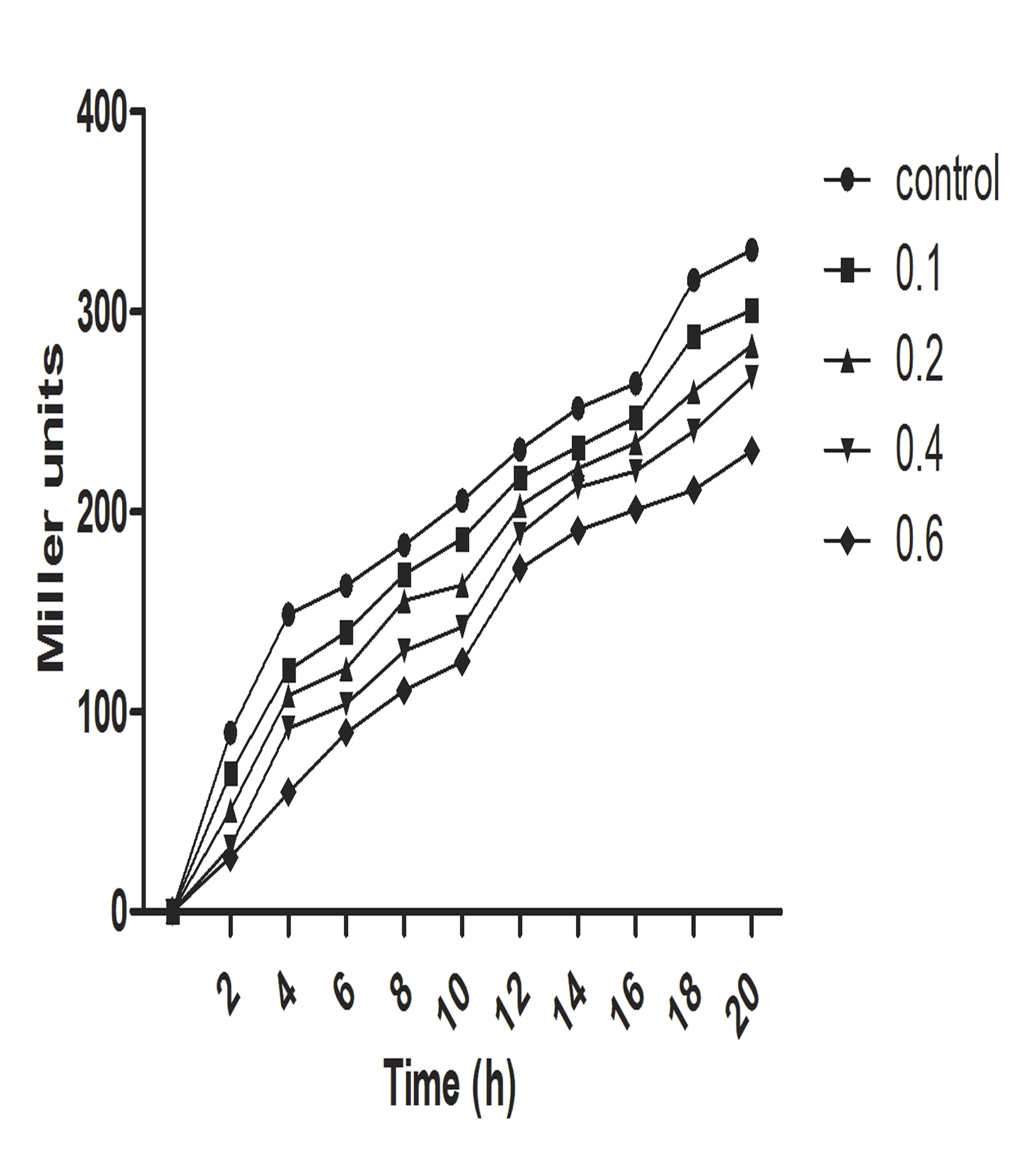

Supplement: S1 Fig — (TIF) [file pone.0135495.s001.tif]

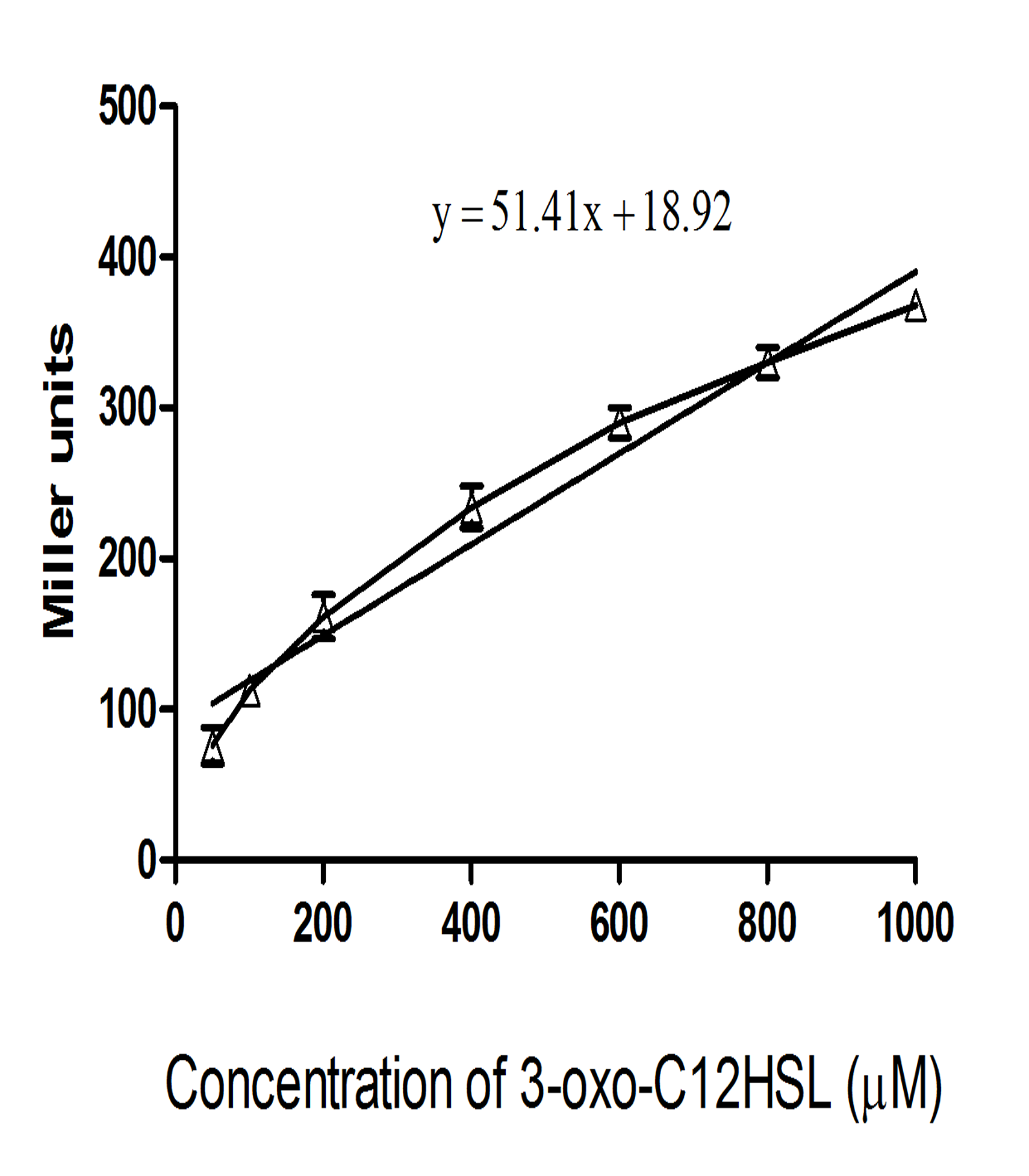

Supplement: S2 Fig — (TIF) [file pone.0135495.s002.tif]
